# Epigenetics in ovarian cancer: premise, properties, and perspectives

**DOI:** 10.1186/s12943-018-0855-4

**Published:** 2018-07-31

**Authors:** Qilian Yang, Yuqing Yang, Nianxin Zhou, Kexin Tang, Wayne Bond Lau, Bonnie Lau, Wei Wang, Lian Xu, Zhengnan Yang, Shuang Huang, Xin Wang, Tao Yi, Xia Zhao, Yuquan Wei, Hongjing Wang, Linjie Zhao, Shengtao Zhou

**Affiliations:** 1 Department of Obstetrics and Gynecology, Key Laboratory of Birth Defects and Related Diseases of Women and Children of MOE and State Key Laboratory of Biotherapy, West China Second University Hospital, Sichuan University and Collaborative Innovation Center, Chengdu, 610041 People’s Republic of China; 2 Nanchang University, Nanchang, People’s Republic of China; 3 Sichuan Normal University Affiliated Middle School, Chengdu, People’s Republic of China; 4 Department of Emergency Medicine, Thomas Jefferson University Hospital, Philadelphia, USA; 5 Department of Surgery, Emergency Medicine, Kaiser Santa Clara Medical Center, Affiliate of Stanford University, Stanford, USA; 6 Department of Biomedical Sciences, City University of Hong Kong, Kowloon Tong, Hong Kong China; 7 Department of Pathology, West China Second University Hospital, Sichuan University, Chengdu, People’s Republic of China

**Keywords:** Ovarian cancer, Epigenetics, Histone methylaiton, Histone acetylation

## Abstract

Malignant ovarian tumors bear the highest mortality rate among all gynecological cancers. Both late tumor diagnosis and tolerance to available chemical therapy increase patient mortality. Therefore, it is both urgent and important to identify biomarkers facilitating early identification and novel agents preventing recurrence. Accumulating evidence demonstrates that epigenetic aberrations (particularly histone modifications) are crucial in tumor initiation and development. Histone acetylation and methylation are respectively regulated by acetyltransferases-deacetylases and methyltransferases-demethylases, both of which are implicated in ovarian cancer pathogenesis. In this review, we summarize the most recent discoveries pertaining to ovarian cancer development arising from the imbalance of histone acetylation and methylation, and provide insight into novel therapeutic interventions for the treatment of ovarian carcinoma.

## Background

Malignant ovarian tumor has the highest mortality rate among all gynecological cancers [1]. Epithelial ovarian cancer (EOC) is the most common type of ovarian cancer [2]. In general, the majority of EOC patients are diagnosed in advanced stage (Stage III or IV) disease, due to the non-specific symptoms characteristic of early stage EOC and the lack of available EOC-specific screening biomarkers. Standard EOC therapy consists of debulking surgery followed by platinum-based chemical therapy [3, 4]. While the initial tumor response is frequently promising, unfortunately, tumors recur rapidly due to chemo-resistance. Acquired chemo-resistance is a daunting challenge in EOC treatment [5]. Thus, the identification of novel cancer-specific biomarkers capable of detecting early stage disease, as well as efficient therapeutic agents against EOC recurrence, is vital for EOC treatment.

**Fig. 1:**
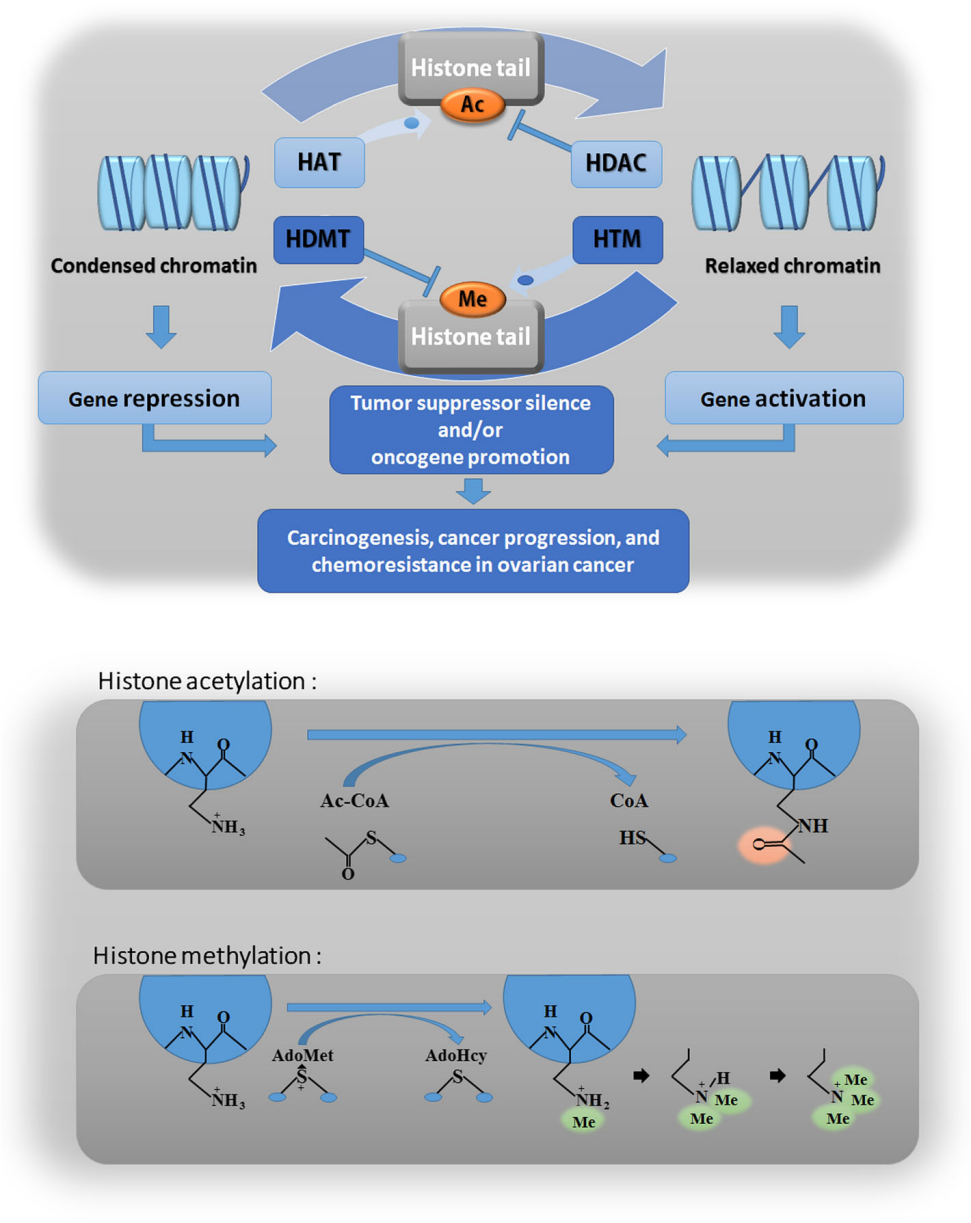
A schematic mechanism of histone acetylation and methylation. The balance of histone acetylation and methylation is respectively controlled by histone acetyltransferases-deacetylases and histone methyltransferases-demethylases. Acetylation of histone tails is associated with a relaxed chromatin structure and transcriptional activation. Conversely, methylation of histone tails is linked with a condensed chromatin structure and transcriptional suppression. Disruption of the steady state of histone acetylation and methylation may cause abnormal cellular function, possibly even ovarian cancer

The genesis of cancer lies within gene alteration [6]. However, epigenetics (the phenotypic alteration in gene expression without modification of the DNA sequence itself) has increasingly been recognized for its role in tumor formation. Recently, a series of cancer-associated genes, regulated by epigenetic modification, has been implicated in the onset and progression of malignant ovarian tumor [7]. Epigenetics includes DNA methylation, nucleosome repositioning, histone post-translational modification, and post-transcriptional gene regulation by miRNAs [8]. Specifically, histone modification, regulated by histone modifying enzymes, manipulates gene expression [9, 10]. Histone modification alters chromatin structure, and is heritable, passing to daughter cell generations. The basic building blocks of chromatin, each nucleosome harbors an octameric core, assembled by two copies of each histone (H2A, H2B, H3, and H4) protein wrapped by 145–147 DNA base pairs (bp). The linker histone H1 binds nucleosomes together, which fold into higher-order chromatin structures [11]. The amino-terminal tails of histones are flexible and unstructured, capable of directly altering the structure of chromatin and ultimately affecting gene expression via enzyme modification. Histone modification dynamically maintains the steady state of chromatin. At least eight different histone modifications exist: acetylation, methylation, phosphorylation, ubiquitination, glycosylation, sumoylation, ADP-ribosylation, and carbonylation [12, 13]. The balance of histone acetylation and methylation is respectively controlled by histone acetyltransferases-deacetylases and histone methyltransferases-demethylases. Disruption of the steady state may cause abnormal cellular function, possibly even ovarian cancer (Fig. 1) [14, 15].

## Potential biomarkers of histone modifications for ovarian cancer

Decades of study have detected a variety of biomarkers for diagnosis and prognosis of ovarian cancer. The most widely used is the biomarker CA125 (also known as mucin 16). The CA125 blood test is not an effective screening test when used alone, given that CA125 levels are only increased in 50% of stage I ovarian cancers and can also be increased in benign disorders, such as uterine fibroids, ovarian cysts and other conditions such as liver disease and infections [16, 17]. Increased levels of CA125 are most frequently observed in high grade serous carcinoma(HGSC), with lower levels of CA125 in other non-serous subtypes. The combination of the CA125 blood test and radiographic imaging, such as transvaginal ultrasonography, has been evaluated for use as a screening strategy. One of the largest studies to examine this combination was the PLCO Cancer Screening trial, which enrolled 78,216 women 55–74 years of age [18]. Ovarian cancer was diagnosed in 212 women (5.7 per 10,000 person-years) in the screening group and in 176 women (4.7 per 10,000 person-years) in the usual care group (rate ratio: 1.21; 95% CI: 0.99–1.48), and the stage distributions of cancer were similar for the two groups (stage III and stage IV cancers comprised almost 80% of cancers in both groups). Although the CA125 test alone as a screening marker has been considered ineffective, the UKCTOCS study evaluated longitudinal measurements of CA125 levels for the screening of ovarian cancer in an algorithm termed ‘risk of ovarian cancer algorithm’ (ROCA) [19]. The mortality reduction was not significant between any of the research groups in this trial and thus, the ROCA test cannot currently be recommended as a screening strategy for ovarian cancer; further follow-up of this study is necessary to understand the long-term potential of this screening strategy. Another ovarian cancer biomarker is human epididymis protein 4 (HE4; also known as WFDC2) [20]. A systematic review reported better sensitivity, specificity and likelihood ratios for HE4 compared with CA125, but this has not yet been analysed within a screening strategy [21]. The use of other novel markers for ovarian cancer screening are under investigation, including, for example, DNA analysis of uterine lavages or Pap smears for TP53 mutations [22]. In this sense, currently none of these biomarkers could be used as an exact index for diagnosis and prognosis prediction of ovarian cancer patients due to the lack of sensitivity and specificity. Despite the mechanisms of how these histone modification alterations arise in ovarian cancer remain unclear, the fact that aberrant histone modification occurs frequently in malignant ovarian tumors and are thought to contribute both to the initiation and development of ovarian cancer. Hence, the exploration of histone modification holds great promise in revealing attractive biomarkers for diagnosis, prognosis and therapeutic targets in women with malignant ovarian tumors.

## Histone acetylation

In general, histone acetylation relates to the relaxed chromatin state, which facilitates gene transcription. The overall level of histone acetylation is a dynamic process, controlled by two opposing enzymes: histone acetyltransferases (HATs) and histone deacetylases (HDACs) [23]. These enzymes have a profound effect upon the structure of chromatin. Imbalance between HATs and HDACs contributes to the pathogenesis of ovarian cancer [15].

### Histone acetyltransferases (HATs)

Utilizing acetyl coenzyme A (acetyl-CoA) as a common acetyl donor, HATs catalyze the transfer of the acetyl group to the ε-amino group of lysine side chains. This action directly abolishes the positive charge of lysine, eliminating the electrostatic bond between DNA and histone [23]. HATs, therefore, unfold the local chromatin structure, rendering it more accessible to non-chromatin proteins.

### Classification and biology of HATs

**Fig. 2:**
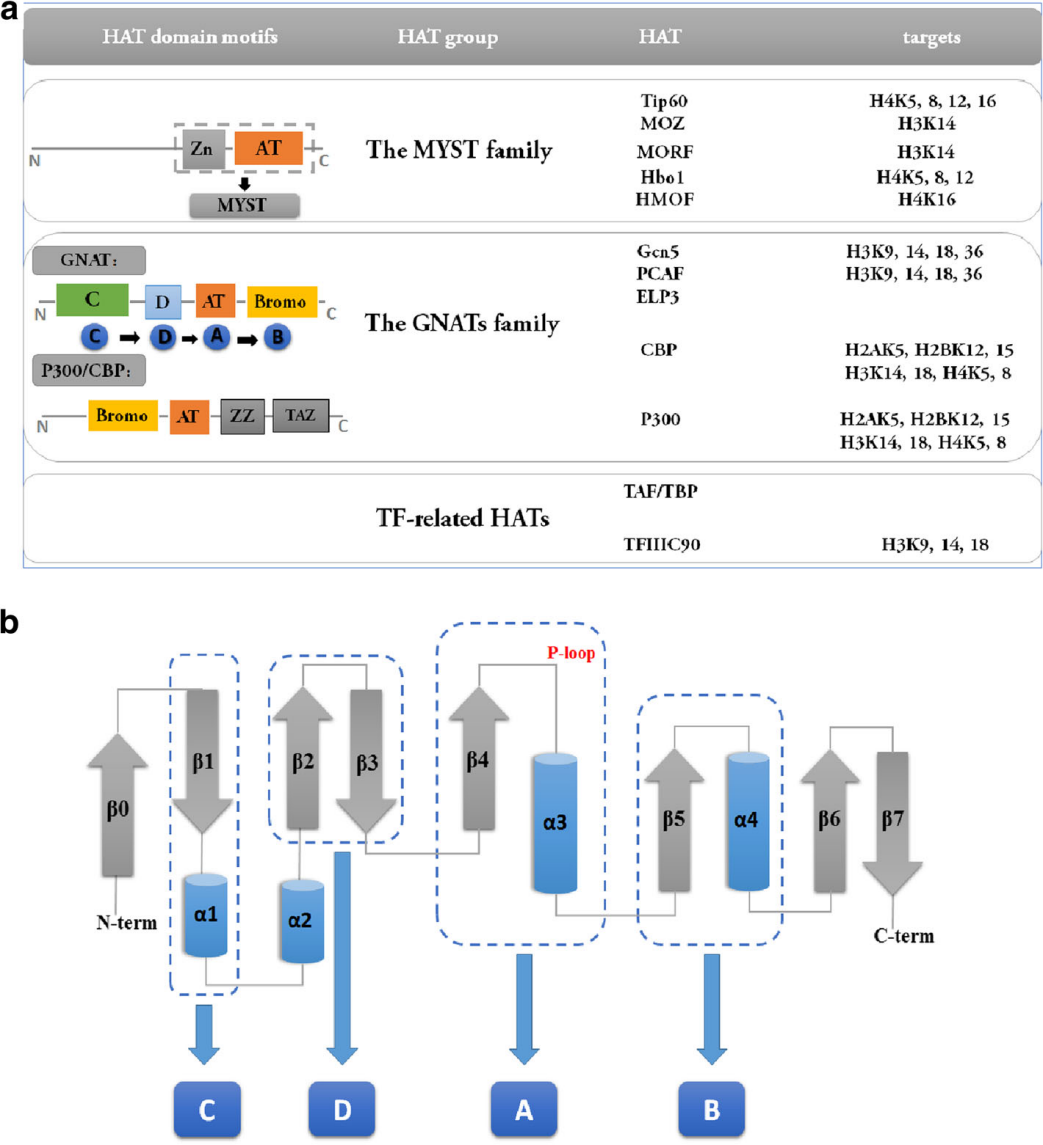
**a** Type A HAT families. AT: acetyltransferase domain; Zn: Zic finger domain; Bromo: bromodomain. GNAT domain includes three key motifs and these motifs in structure is in this order: D-A-B. Some of GNAT family members contain another conserved motif C that is located at the N-terminus of these proteins**. b** Topological diagram of the core GNAT fold. The highly conserved GNAT domain composed of 6–7 anti-parallel β-strands and 4 α-helices in the topology β1-α1-α2-β2-β3-β4-α3-β5-α4-β6-β7. Motifs A and B participate in acyl-CoA and acceptor substrate recognition and binding, and the feature of motif A is the “P-loop” that connect helix α3 and strand β4. Motifs C and D preserve the stability of proteins

Two major HAT types exist: nuclear (A-type) and cytoplasmic (B-type) (Fig. 2) [24]. Type-A HATs are categorized by structural homologies and functional similarities into three subfamilies: the MYST (Moz-Ybf2/Sas3-Sas2-Tip60) family, the GCN5-related N-acetyltransferases (GNAT) family and the p300/CREB-binding protein (CBP/CREBBP) family. The MYST family contains a specific sequence region (called MYST domain), made of an acetyl-CoA binding motif and a zinc finger. Some MYST family members also possess additional structural features termed chromo-domains (MOF, Esa1, and Tip60) [25]. The GNAT enzyme superfamily is widespread in nature [26]. In spite of substrate diversity, GNAT superfamily members are generally characterized by a highly conserved GNAT domain (composed of 6–7 anti-parallel β-strands and 4 α-helices in the topology β1-α1-α2-β2-β3-β4-α3-β5-α4-β6-β7 [26, 27]), and can acetylate lysine residues on histones H2B, H3, and H4. GNAT domain contains three conserved motifs in the order of D-A-B. Motifs A and B are highly conserved, and participate in acyl-CoA and acceptor substrate recognition and binding. Motifs D preserves the stable state of these proteins. Some other members of GNAT family harbor another conserved motifs C that is located at the N-terminus of these proteins [27, 28]. Although the structure of p300/CBP family has not yet been fully elucidated, several studies have shown that the structure and the catalytic mechanism of this group of proteins are obviously distinct from the MYST and GNAT families [29]. Except for a large HAT domain (about 500 residues) and a bromodomain, most importantly, the p300/CBP family possesses three putative zinc finger domains (ZZ, PHD and TAZ) that mediate protein-protein interaction, as well as two protease-resistant domains connected by a long protease-sensitive loop, which are not seen in other HAT members [28–30]. Moreover, it has been observed that the p300/CBP family employs a Theorell-Chance, or “hit” and “run” mechanism which differ from the mechanism of GNAT proteins (a sequential mechanism) and MYST proteins (a ping pong mechanism) [29]. Hat1 (histone acetyltransferase 1), which was the first histone acetyltransferase identified, is the only representative member of B-type HATs [31]. This enzyme is primarily located in the cytoplasm. It acetylates free (not nuclear) histones. Interestingly, Hat1 is also found in the nucleus [31]. Type-B HATs acetylate newly synthesized histone H3 and H4 (Type-B HATs are mainly responsible for newly synthesized H4K5 and H4K12). Newly synthesized histones H3 and H4 are rapidly acetylated and then the modifications of acetylation are removed after the assembly of the histones into nucleosomes in the mature process of chromatin [31, 32].

### The role of HATs in ovarian cancer

Human males lack hMOF (also known as MYST1, a member of the MYST family of HATs), the human ortholog of the Drosophila MOF protein [33]. MOF contains a chromodomain and acetyl-CoA binding motif. Biochemical purifications reveal hMOF forms two distinct multicomplexes in mammalian cells: MSL (Male Specific Lethal)-associated MOF and NSL (Non-Specific Lethal)-associated MOF [34]. MSL-associated MOF exhibits strong specificity for histone H4K16 [27, 35]. NSL-MOF exhibits relaxed substrate specificity [36], and acetylates histone H4 at K5, 8, and 16 [27, 34, 37, 38]. Depletion of hMOF reduces histone H4K16 acetylation, gene instability, and cell cycle disorder. Although the specific mechanisms hMOF plays in tumor development and progression are unclear, several studies demonstrate that abnormal hMOF gene expression in various cancers, including breast, renal cell, colorectal, gastric, and non-small cell lung cancer [39–42]. Recent studies demonstrated that HCP5 (human leukocyte antigen (HLA) complex 5) is a target gene of hMOF, and marked down-regulation of hMOF and HCP5, and loss of H4K16 acetylation were observed in ovarian epithelial cancer tissues [38, 43]. Immune system, as we known, prevents or controls tumor by monitoring cell biological behavior and identifying and eliminating abnormal cells. The aberrant expression of immune molecules like HLA-class I and II has been found in ovarian cancer (The human leukocyte antigen (HLA) system or complex is a gene complex encoding the major histocompatibility complex (MHC) proteins in humans) [44]. Moreover, HCP5 is localized within the MHC class I region and plays a key role in immunity to retrovirus infection [45]. Therefore, hMOF may have a role in modulation of tumor antigen-specific immune responses in ovarian cancer through modulating the expression of its target gene HCP5. Decreased hMOF levels are associated with reduced overall patient survival [43]. As such, hMOF protein expression is an independent risk factor influencing malignant ovarian tumor prognosis [38, 43]. hMOF may therefore have value as both a novel epigenetic biomarker for the diagnosis of malignant ovarian tumor, as well as a target for EOC treatment.

### Histone deacetylases (HDACs)

HDACs remove acetyl residues, restoring the positive charge of lysine. Consequently, HDACs are associated with condensed chromatin structures and transcriptional repression [23].

### Classification and biology of HDACs

**Fig. 3:**
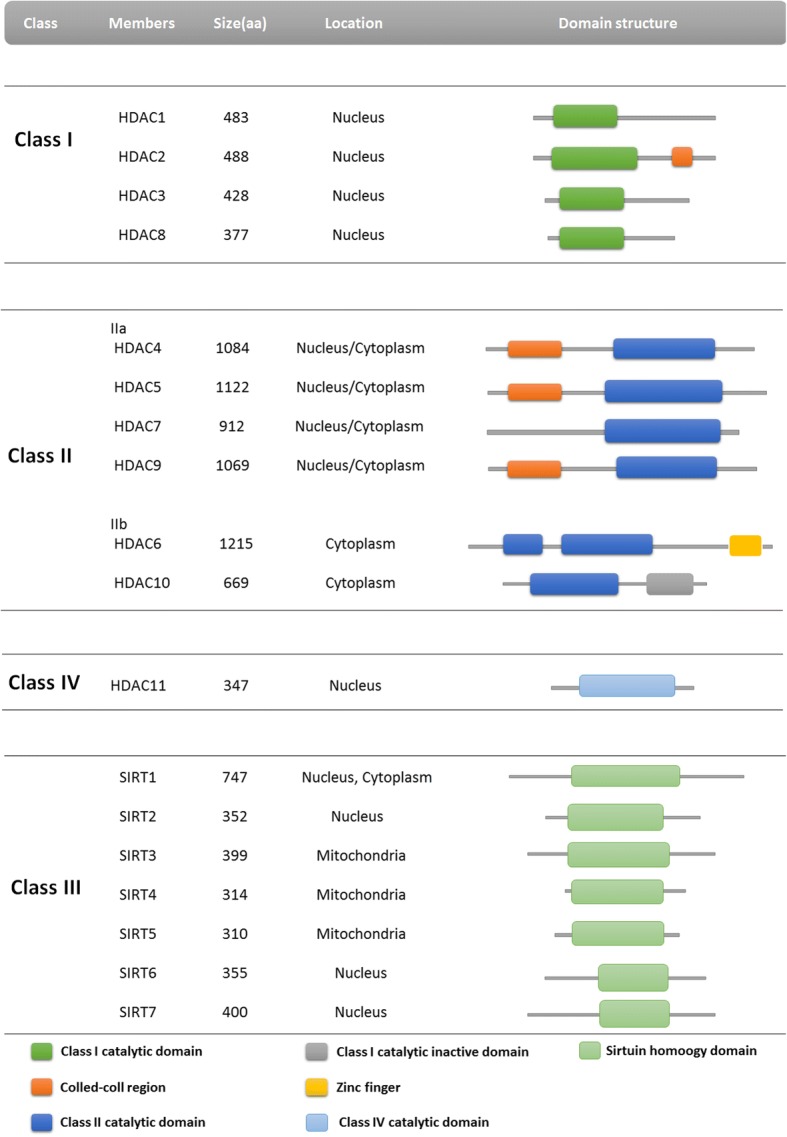
HDAC families and their domain structure

Heretofore, the family of HDACs includes 18 isoenzymes, sorted into four classes (I-IV) based upon homology to yeast HDACs and sequence similarity (Fig. 3) [46]. Classes I, II, and IV are zinc-dependent enzymes. Class III (also known as sirtuins/SIRTs) are NAD+ dependent enzymes. Class I HDACs include HDAC 1, 2, 3, and 8 (all nuclear proteins). Of these, HDAC3 shuttles between the cytoplasm and nucleus. Class II HDACs include HDAC 4, 5, 6, 7, 9, and 10. All Class II HDACs shuttle between the cytoplasm and the nucleus. Class II consists of two subfamilies: IIa (HDAC 4, 5, 7, and 9) and IIb (HDAC 6 and 10). Subfamily IIa possess a highly conserved 600-residue long N-terminal extension. The N-terminal extension possesses sites that bind myocyte enhancer factor 2 (MEF2) and 14–3-3 proteins. HDAC11 is the sole member of Class IV HDACs and share properties with both Class I and II. Class III HDACs are mammalian homologs of the yeast silent information regulator (SIR2), and include seven members (SIRT1–7) with possessing distinctive targets. Class III HDACs act as deacetylases for histones and non-histones [47–49].

### The role of HDACs in ovarian cancer

#### Classical HDACs

##### Class I (HDAC 1, 2, and 3)

Class I HDACs (1, 2, and 3) promote ovarian cancer progression. Class I HDACs are over-expressed in ovarian cancer tissues, and play a critical role in ovarian carcinogenesis [50]. Moreover, expression of class I HDACs increases gradually from benign, borderline, and malignant ovarian tumors. Class I HDAC expression levels are markedly different in various histological ovarian cancer subtypes. Class I HDAC expression is most positive in mucinous subtypes, followed by high-grade serous, clear cell, and endometrioid subtypes. Strongly proliferating tumor tissues exhibit increased Class I HDAC expression. In addition, increased Class I HDAC expression is an independent risk factor for poor malignant ovarian tumor prognosis [50, 51]. The specific mechanisms underlying how Class I HDACs facilitate ovarian carcinogenesis and chemo-resistance remain incompletely understood. Recently, a study further demonstrates the downregulatiom of RGS2 in drug-resistant ovarian cancer cells partly because Class I HDACs suppress the promoter region of RGS2 [52]. RGS2 (Regulator of G-protein Signaling 2) is an inhibitor of G-protein coupled receptors (GPCRs) via accelerating the deactivation of heterotrimeric G-proteins. The level of RGS2 dropped sharply in ovarian epithelial cells resistant to chemotherapy compared with chemo-sensitive cells has been observed [53]. HDAC1 enhances cellular proliferation via of cyclin A promotion [54]. HDAC2 remodels chromatin in response to platinum-based chemical therapies in ovarian epithelial cancer cells [15]. HDAC3 facilitates cellular migration by suppressing E-cadherin expression [54].

Interactions between the immune system and tumor critically impact prognosis. Recently, it has been demonstrated that ovarian cancer generates an immunosuppressive microenvironment to evade immune system attack. Proteins OX-40 ligand (OX-40 L/TNFSF4/CD134L/CD252) and 4-1BB ligand (4-1BBL/TNFSF9/CD137L) regulate effector cytotoxic T-cell (CTL) activity while programmed death ligand-1 (PD-L1) exhibits immunosuppressive effects, allowing the tumor to escape immune destruction [55]. Drug-resistant ovarian cancer cells exhibit repression of OX-40 L and 4-1BBL (immune-stimulatory molecules), with concomitant augmented expression of immunosuppressive molecules PD-L1/CD274 [56]. Furthermore, HDAC1 and HDAC3 exhibit aberrant association with OX-40 L and 4-1BBL promoters in chemotherapy-resistant ovarian cancer cells, contributing to suppression of OX-40 L and 4-1BBL [56]. EOC is one of the first malignancies demonstrating correlation between tumor-infiltrating lymphocytes (TILs) and increased overall survival rate [55, 57, 58]. Moreover, two HDAC1/2-derived HLA ligands activate T-cells, prompting further elimination of HLA-matched cancer cells [59]. HDAC1 and HDAC7 maintain cancer stem cells (CSCs), both of which are over-expressed in ovarian cancer CSCs compared to non-stem tumor cells (NSTCs) [60]. Additionally, accumulation of HDAC4 (generated by nuclear fibrillar collagen matrices by PP1α co-localization) suppresses p21, facilitating ovarian cancer cell proliferation, increasing invasive potential, and promoting migration [61].

#### HDAC10

The function of histone deacetylase 10 (HDAC10, a Class IIb member) in EOC is poorly understood. HDAC9 and HDAC10 are required for homologous recombination [62]. Recent evidence suggests HDAC10 inhibitors may augment platinum therapy efficacy in malignant ovarian tumors [63].

#### SIRTs

Yeast SIR2 (silent information regulator 2, member of the sirtuin family) was originally isolated during screening for cancer silencing factors [64]. SIR2 is a nicotinamide adenine dinucleotide (NAD+)-dependent enzyme, and is a histone deacetylase [64–66]. It is implicated with calorie restriction associated life span extension [67, 68]. Heretofore, 7 mammalian homologues (SIRT1–7) have been defined, with SIRT1 closest evolutionarily to yeast SIR2. Mammalian sirtuins target different sites, have diverse substrates, and influence various cellular functions.

#### Sirtuin-1 (SIRT1)

**Fig. 4:**
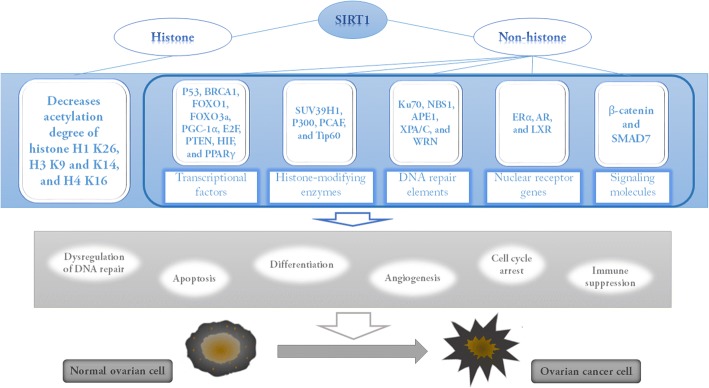
A series of targets of SIRT1, and its multiple pathways that contribute to ovarian cancer. SIRT1 deacetylases both histones and non-histones. SIRT1 could directly decrease the degree of acetylation of histone H1K26, H3K9, H3K14, and H4K16, and also indirectly regulate the methylation and acetylation of histone by interacting with other histone-modifying enzymes such as SUV39H1, P300, PCAF, and Tip60. The non-histone substrates of SIRT1 (transcriptional factors, DNA repair machinery elements, nuclear receptor genes, and signaling molecules) are critical in the initiation and progression of ovarian cancer

Of the 7 sirtuin family members, SIRT1 is among the most studied. SIRT1 has the highest homology to Yeast SIR2 [68]. SIRT1 protects against DNA damage and genomic instability, as well as cellular oxidative stress [69–71]. SIRT1 deacetylases both histones and non-histones (Fig. 4), and directly decreases the degree of acetylation of histone H1 K26, H3 K9, H3 K14, and H4 K16 [65, 72]. SIRT1 indirectly regulates histone methylation by interacting with methyltransferase SUV39H1 during heterochromatin formation [73]. The non-histone substrates of SIRT1 (transcriptional factors, DNA repair machinery elements, nuclear receptor genes, and signaling molecules) are critical in various biological processes, but are particularly important in carcinogenesis [74–77]. SIRT1 mediates deacetylation of p53, allowing cells with damaged DNA to bypass cell-cycle control, enabling mutation accumulation and, ultimately, carcinogenesis [78]. It may contribute to EOC development. SIRT1 is markedly increased in malignant ovarian tumors (especially, in serous carcinoma) compared to corresponding normal tissues, and up-regulated SIRT1 inactivates p53 by deacetylation [79]. SIRT1 overexpression is more common in early stage EOC [79]. EMT (epithelial-to-mesenchymal transition) occurs during early to invasive stage phenotypic tumor transition. SIRT1 regulates EMT in ovarian cancer cells, crucial in ALS-induced autophagy, antagonizing hypoxia-induced EMT [80]. SIRT1 is also implicated in LPA (lysophosphatidic acid)-induced EMT in ovarian cancer cells [59]. Ovarian cancer cell resistance to platinum-based drugs is largely attributed to increased SIRT1 expression [79, 81, 82]. The tumor suppressor gene BRCA1 regulates SIRT1 expression and NAD activity [83]. Crosstalk between SIRT1 and BRCA1 has been demonstrated but via unclear precise mechanisms. SIRT1 modulates cisplatin sensitivity in ovarian cancer via BRCA1-SIRT1-EGFR signaling [83, 84]. SIRT1 over-expression is pivotal in malignant ovarian tumor chemo-resistance, and may serve as a predictive indicator of poor clinical outcome [85].

#### Sirtuin-3 (SIRT3)

The Class III HDAC member SIRT3 functions as a malignancy inhibitor or facilitator dependent upon cancer type [86]. SIRT3 inhibits ovarian cancer cell migration via Twist down-regulation [87]. SIRT3 is requisite in the function of Bcl-2 suppressors in EOC, regulating both S1-mediated glucose metabolic and apoptotic effects [88].

#### Sirtuin-6 (SIRT6)

SIRT6 has been implicated in the development of colon adenocarcinoma, pancreatic, breast, and liver cancer [89–91]. As a tumor suppressor, SIRT6 is downregulated in ovarian cancer. SIRT6 inhibits ovarian cancer cell proliferation via Notch3 downregulation, and correlates with ovarian carcinoma prognosis [92].

## Histone methylation

Histone methylation mainly occurs upon lysine or arginine residues. Methylation is required for various biological processes, ranging from post-transcriptional regulation to faithful chromosomal transmission during mitosis [93–95].

Unlike acetylation, however, histone methylation cannot alter histone protein charge [96]. Histone lysine methylation modulates either transcriptional activation (e.g. H3K4me1/me2/m3, HK36me3, H3K79me1/me2/me3, H4R3me1, H4K20me1) or gene silencing (e.g. H3K9me2/me3, H3K27me3) depending upon 1) the particular residue methylated, 2) the degree of methylation, and 3) the site of the methylated histone within a specific gene locus. Moreover, histone methylation has stronger location-specificity than histone acetylation that generally links with transcriptional activation [97–99]. Histone modification leads to different biological consequences due to recruitment of diverse effector proteins [98, 100]. The steady state of histone methylation is maintained by a balance between histone methyltransferases (HMTs) and histone demethylases (HDMTs). HMTs add methyl groups to the side chains of lysine and arginine. HDMTs catalyze methyl group removal. Therefore, imbalance between HMTs and HDMTs leads to aberrant gene expression, and carcinogenesis may ensue, including ovarian cancer [101–104].

### Histone methyltransferases (HMTs)

HMTs are a large family of protein methyltransferases, adding methyl groups to lysine (HKMTs) or arginine (PRMTs). Both utilize SAM (S-Adenosyl-l-Methionine) as a methyl group donor. Lysine can be mono-, di-, or tri-methylated. Arginine residues of the core or tails of histones can be mono- or di- (asymmetric or symmetric) methylated [105].

### Classification and biology of HMTs

#### Lysine methyltransferases (HKMTs)

**Fig. 5:**
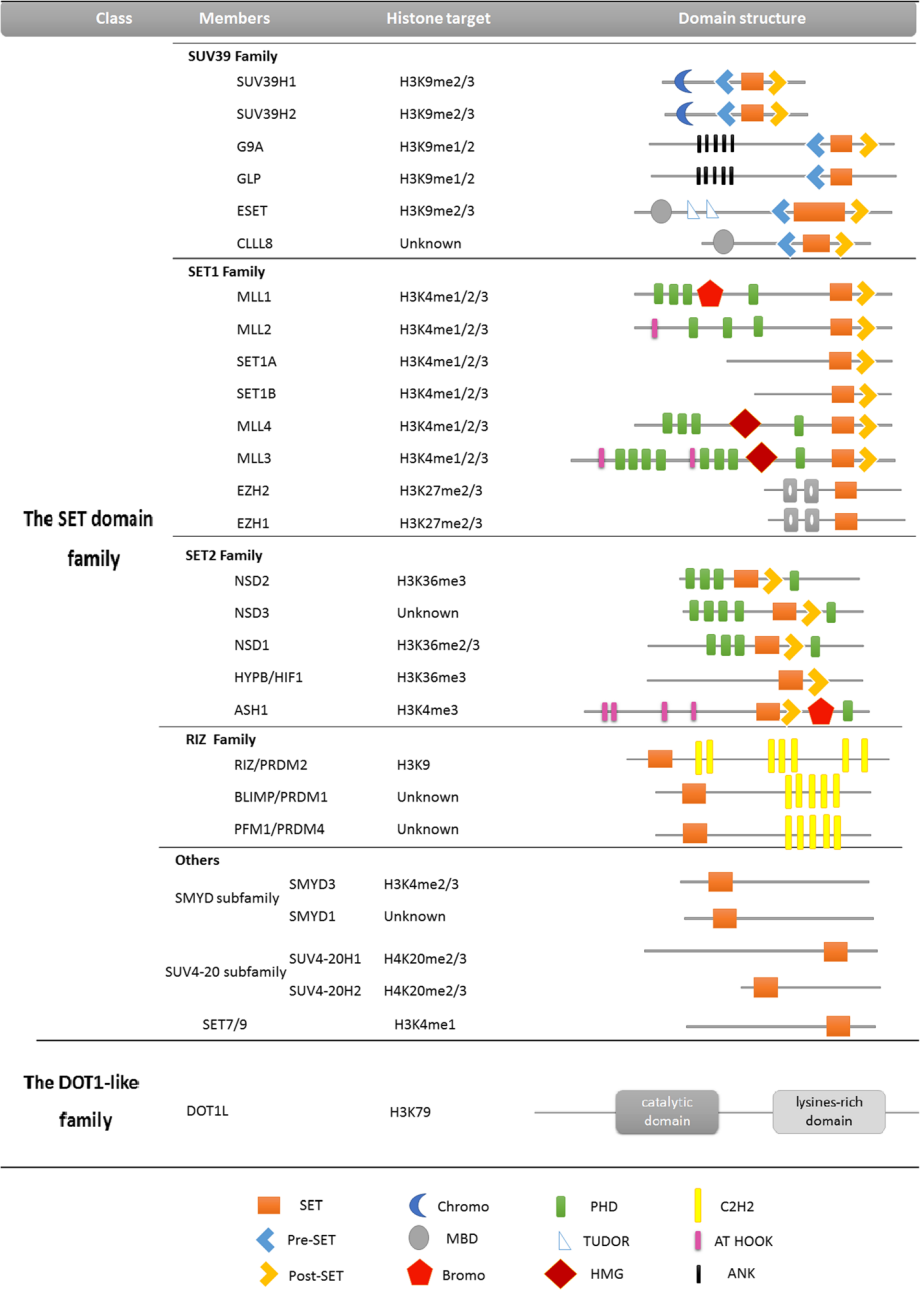
Histone lysine methyltransferases(HKMTs): classfication, histone targets, primary domain architecture. HKMTs are classified in two types: the SET domain-containing proteins and the DOT1-like proteins. The SET domain-containing proteins can be subdivided into four families by structural sequence features: SUV39, SET1, SET2 and RIZ. Except for these family listed above, there are other SET domain -containing methyltransferases that have not been classfied into a specific group, for instance, SET8, SET7/9, SMYD subfamily and SUV4–20 subfamily

HKMTs are very sensitive and specific to the histone lysine residue they target, as well as the degree of methylation they can perform. Most lysine methyltransferases possess an evolutionarily conserved SET domain, referring to a multiprotein complex first identified in the Drosophila polycomb group proteins, namely suppressor of variegation 3–9 (Su(VAR)3–9), enhancer of zeste (E(z)), and trithorax (TRX) (Fig. 5) [106]. Lysine methyltransferases usually function within this multiprotein complex. The SET methyltransferase is responsible for the catalytic domain, while the others complex components account for selectivity and activity [107]. Centromeric heterochromatin is characterized by tri-methylated H3K9. Methylated H3K9 within the centromeric heterochromatin is requisite for recognition and binding of HP1 (heterochromatin protein 1). Suv39 loss directly induces down-regulation of H3K9 tri-methylation, influencing mitosis and meiosis [108]. Some SET demethylases are classified into subfamilies by structural sequence features: SET1, SET2, SUV39, EZ, RIZ, SMYD, and SUV4–20 subfamilies. Other SET domain-containing methyltransferases have not been classified into a specific group [107]. Additionally, the DOT1 (disruptor of telomeric silencing)-like family specifically methylates H3K79 at the histone globular core, maintaining meiosis stability. It does not contain the SET domain [109].

#### Arginine methyltransferases (PRMTs)

The multiple arginine residues within histone tails are mono- and di-methylated, affecting nucleosome remodeling and gene expression. Methylarginines have three different forms: Mono-methylated arginine (MMA), symmetric di-methylated arginine (SDMA), and asymmetric di-methylated arginine (ADMA) [110, 111]. There are nine known PRMTs in mammals, which are grouped into three classes: type-I, type-II, and type III enzymes. The type-I enzymes catalyze mono- and asymmetric di-methylation of arginine residues, and they include PRMT1, 2, 3, 4 (also known as CARM1), 6, and 8. The type-II enzymes catalyze mono- and symmetric di-methylation of arginine residues (PRMT5 and PRMT9 fall into this category). The type-III enzymes exclusively catalyze mono-methylation of arginine residues and only includes PRMT7 [111–113]. All of these enzymes catalyze the transfer of the methyl group to the guanidine nitrogen atom of arginine residues in a variety of histone, non-histone proteins, and various substrates. The most relevant enzymes in histone arginine methylation are PRMT1, 4, 5 and 6.

### The role of HMTs in ovarian cancer

#### EZH2

**Fig. 6:**
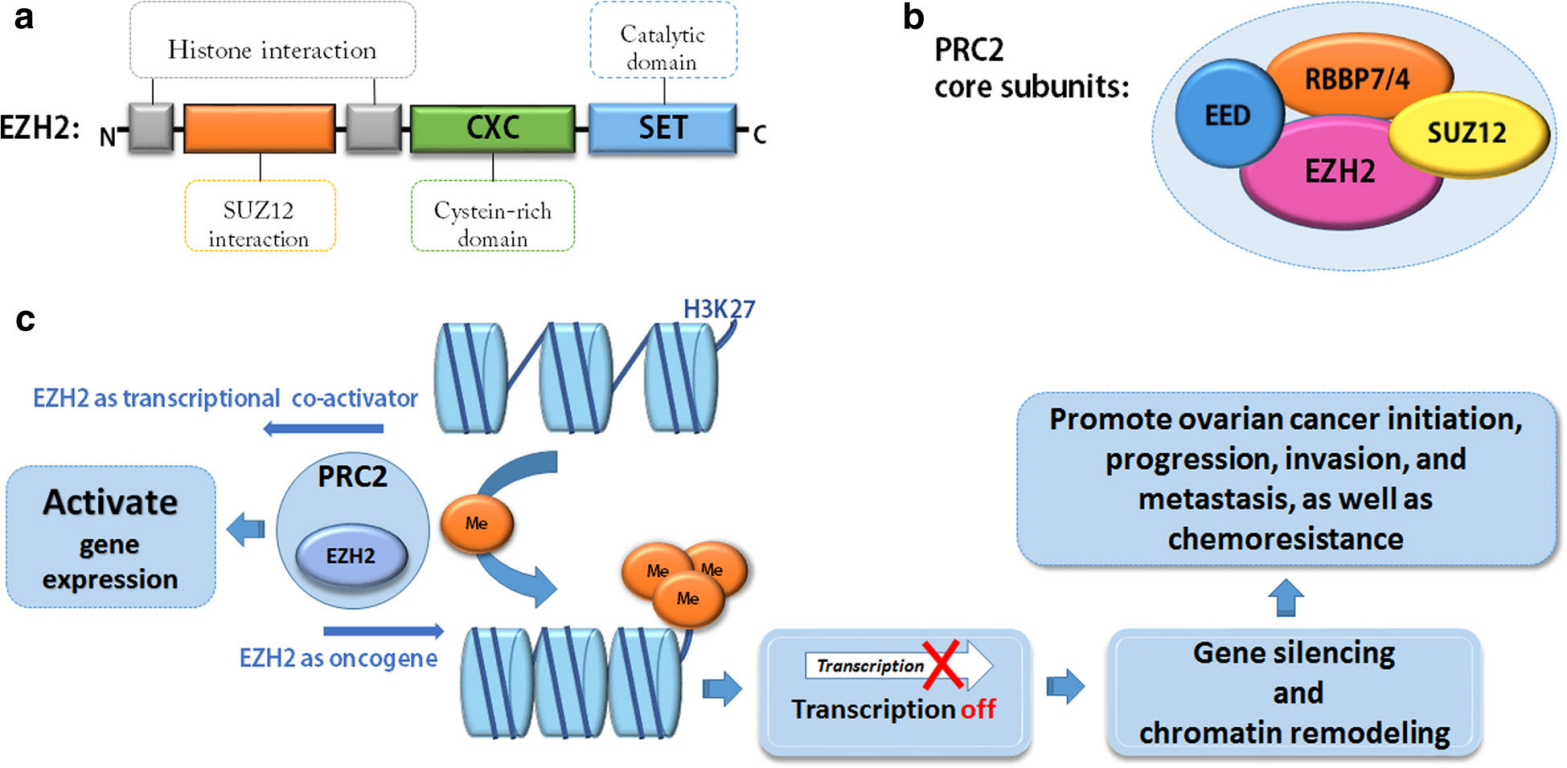
The structure and function of EZH2. a. The schematic view of EZH2 principal domain; **b**. Four core subunits of PRC2; **c**. The schematic illustration of EZH2 function in ovarian cancer

Enhancer of zeste homologue 2 (EZH2), a member of SET1 family, is an integral subunit of the polycomb repressive complex 2 (PRC2) and possesses histone methyltransferase activity on lysine-9 and -27 of histone 3 or lysine-26 of histone 1 (Fig. 6). EZH2 is primarily responsible for H3K27 methylation, and the tri-methylated H3K27 (H3K27me3) is correlated with the gene silencing [114, 115]. Additionally, the polycomb group protein EZH2 can also serve as a platform to recruit DNA methyltransferases and further directly control the methylation states of DNA [116]. The mammalian PRC2 complex mainly contains four core components: EZH2 (catalytic core component of PRC2), EED (Embryonic Ectoderm Development), SUZ12 (Zinc finger protein suppressor of Zeste 12), and RbAp46/48 [117, 118]. Different forms of the EZH2 complex exist in cells, which are distinguished by the different N-terminal lengths of EED contained [118]. EZH2 is known to silence gene expression via trimethylation of histone H3 on lysine 27 (H3K27me3) [119]. However, recent evidence implicates a PRC2-independent role of transcriptional activation for EZH2. In a castration-resistant prostate cancer model, EZH2 acted as a co-activator for critical transcription factors including the androgen receptor (AR) that was independent of its transcriptional repressor function [120]. Moreover, EZH2 physically bridged the estrogen receptor (ER) and components of Wnt signaling to induce the gene expression in breast cancer cells [121]. EZH2 also activated NF-κB targets of NOTCH1 in breast cancer cells [122].

The expression of EZH2 is upregulated in many carcinomas, with the high level of EZH2 correlated with poor outcome of human tumors. EZH2 is involved in tumor initiation, development, progression, metastasis and chemoresistance through gene silencing and chromatin remodeling [123]. EZH2, as an oncogene, mainly functions to inhibit the expression of tumor suppressor genes through upregulating their methylation level. There is mounting evidence that up-regulation of EZH2 occurs in ovarian carcinoma, and is positively correlated with worsening histological grade and advanced stage [124]. EZH2 is thought to be an independent forecaster of poor overall survival for women with ovarian carcinoma [125–127]. Breast cancer 1 (BRCA1) gene is a well-recognized tumor suppressor gene, and the loss of BRCA1 is closely associated with ovarian carcinoma [128]. Recently, a study has revealed that knockdown of EZH2 can rescue BRCA1 protein expression and facilitate its nuclear translocation. EZH2, therefore, may participates in biological behavior of EOC via modulating the expression of BRCA1 [127]. Moreover, the correlation between EZH2 and transforming growth factor-beta1 (TGF-β1) has also been observed in ovarian carcinoma tissues. EZH2 promotes the ability for invasion and metastasis of ovarian carcinoma cells by regulating TGF-β1 [129]. In detail, Rao et al. demonstrated that in ovarian carcinoma cell lines, EZH2 knockdown was found to reduce TGF-β1 expression and increase E-cadherin expression either in the transcript or in the protein levels. Furthermore, a significant positive correlation between overexpression of EZH2 and TGF-beta1 in ovarian carcinoma tissues was observed, suggesting a potential important role of EZH2 in the control of cell migration and/or invasion via the regulation of TGF-β1 expression.

Moreover, numerous studies support that EZH2 is critical to maintain a stem cell state [125]. The high expression of EZH2 has been implicated in maintenance of the cisplatin-resistant subpopulation of cells in ovarian carcinoma and contributes to acquired-tolerance for platinum-based chemotherapy [126]. Malignant ovarian tumor epigenetics could also be regulated by tumor microenvironment. Recent work has indicated that cancer-associated fibroblasts (CAFs) have an ability to enhance the growth and invasion of ovarian cancer cells, and this ability is partly due to increasing EZH2 expression [130].

#### DOT1L

Disruptor of telomeric silencing-1-like (DOT1L), a human homolog of yeast DOT1, is a unique histone methyltransferase that lacks the SET domain and is only responsible for methylating lysine-79 of histone H3 (H3K79) in the core domain [131]. According to recent findings, DOT1L-mediated H3K79 methylation serves as a key regulator in a number of physiological and pathological processes ranging from gene expression to DNA damage response and cell cycle regulation [132]. Zhang et al. first demonstrated that patients with ovarian carcinoma exhibiting high-level of DOT1L have poorer overall survival and progression-free survival as compared to those with low expression of DOT1L. This observation also suggested that DOT1L can directly regulate the transcription of G1 phase arrest genes CDK6 and CCND3 through H3K79 dimethylation to promote cell cycle progression [133]. The expression of DOT1L can be regarded as an independent predictive factor and a potential area for therapeutic intervention in ovarian cancer.

#### PRMT1

Protein arginine methyltransferase 1 (PRMT1), is an important arginine methyltransferase. It can serve as a transcriptional promotor or inhibitor by modifying a series of various substrates. PRMT1 is linked with many biological processes, including carcinogenesis [134, 135]. PRMT1 is a predominant asymmetric arginine methyltransferase in humans. Asymmetric di-methylation of histone H4 at arginine 3 (H4R3me2as) is mediated by PRMT1 and promotes transcriptional activation [136]. Increasing evidence has connected PRMT1 to the development and progression of cancer. Abnormal expression of PRMT1 has been seen with breast and prostate cancer [137, 138], and may have a role in the progression of ovarian carcinoma. Akter et al. suggest that PRMT1 is critical for the development of ovarian carcinoma. Knock-down of PRMT1 suppresses proliferation, migration, invasion, as well as colony formation of ovarian cancer cells. It has been shown that FAM98A, a new substrate of PRMT1, is expressed in ovarian cancer cell lines and is correlated with migration and invasion of ovarian cancer cells [110].

#### PRMT5

Arginine methyltransferase 5 (PRMT5), the first identified type II arginine methyltransferase [139], is the primary enzyme responsible for mono- and symmetric di-methylation of arginine. PRMT5 localizes to both the nucleus and the cytoplasm and it methylates multiple histone and non-histone proteins [111]. There is growing evidence that PRMT5 is involved in a wide variety of biological processes including cellular differentiation [140], proliferation [141], and apoptosis [142]. PRMT5 can directly methylate H4R3 and H3R8 to silence the tumor suppressor gene ST7 (suppression of tumorigenicity 7) and NM23 (nonmetastatic 23) [111, 143]. Additionally, it also can alter cell biological behaviors by methylating many other substrates, such as P53 [141], E2F1 [142], cyclin E1 [144] and E-cadherin [145]. Evidence has emerged that PRMT5 acts as an oncogene in ovarian cancer. The high level of PRMT5 is expressed in EOC and associated with poor outcome. Furthermore, the finding addresses that PRMT5 could substantially promote growth and proliferation of ovarian carcinoma cells depending on E2F-1 [146]. E2F-1 is a complex family of transcription factors and participates in the regulation of cell proliferation and cell cycle progression [147]. The down-regulation of PRMT5 may be a potential therapeutic target in malignant ovarian tumors. However, the precise role of PRMTs in ovarian cancer has not been elucidated. More studies are needed to explore the work of PRMTs in ovarian cancer.

### Histone demethylases (HDMTs)

It had been thought that the methylation of histone was stable and irreversible, until 2004, when Shi et al. discovered lysine-specific demethylase 1A (LSD1; also known as KDM1A). LSD1 specifically removes methylation from mono- or di-methylated lysine 4 at histone H3 (H3K4me1 and H3K4me2) through its amine oxidase domain [148]. This revelation completely changed the concept of histone methylation.

### Classification and biology of HDMTs

#### Lysine demethylases

Histone lysine demethylases (KDMs) are classified into two categories: the flavin-dependent amine oxidase family and the jumonjic (JMJC) domain-containing family. Although these two families work on the lysine through different catalytic mechanisms, they have similar effects [99, 148].

All members of the amine oxidase-related family belong to the superfamily of the flavin adenine dinucleotide (FAD)-dependent enzymes that form an imine intermediate that is subsequently hydrolyzed to generate unmodified lysine and formaldehyde [149]. During the entire process, the cofactor FAD oxidizes the methyl-lysine and then the reduced FAD is re-oxidized by oxygen. Because the formation of the obligatory imine intermediate in this reaction requires a lone pair of electrons on the methy-lysine ε-nitrogen atom, LSD1 only removes mono−/di-methylation, not tri-methylation [148, 149]. While proteins in this superfamily have a JmjC domain, including six clusters (KDM2 to KDM7), they are a kind of enzymes apparently different from LSD-family, which can utilize Fe^2+^ and α-ketoglutarate (α-KG) as cofactors to remove all 3 lysine methylation states (tri-, di- and mono-methylation) at H3K4, H3K9, H3K27, and H3K36, as well as H1K26 [149]. The JmjC lysine demethylases catalyze protein hydroxylation at the carbon of the Nε-methyl group to produce an unstable hemiaminal intermediate, and Nε-methyllysine demethylation by the hydroxylation [150].

### Arginine demethylases

The human peptidylararginine deiminases 4 (PAD4/PADI4) is capable of transforming mono-methylated histone arginine to citrulline (Cit). However, as methylated arginine and non-methylated arginine can both be demethylated by PAD4/PADI4 and this demethylation reaction produces Cit, rather than arginine, PAD4/PADI4, to be exact, is not a “strict” histone demethylase [151]. In 2007, Chang et al. reported that Jumonji domain–containing 6 protein (JMJD6) is the only known member of the JmjC family that has the arginine demethylase activity and can demethylate histone H3 at arginine 2 (H3R2) and histone H4 at arginine 3 (H4R3) [152]. JMJD6 can catalyze two types of reactions: hydroxylation and demethylation. However, the latter catalytic mechanism is controversial [153]. Thus, the existence of a “true” histone arginine demethylase remains unclear.

### The role of HDMTs in ovarian cancer

#### KDM1 subfamily

Lysine-specific demethylase 1 (LSD1/KDM1A), the first H3K4 lysine-specific demethylase to be recognized [148], is a flavin-containing amine oxidase. It is a highly conserved protein that specifically catalyzes demethylation reactions on mono- and di-methylated histone H3K4 or H3K9. The structure of LSD1 bears three domains. It contains a C-terminal amine oxidase-like domain (AOL), a SWIRM domain located at the N-terminal, and a Tower domain that is inserted into the AOL domain and directly engages the SANT2 domain of CoREST (also known as RCOR1) [154]. The substrate specificity of LSD1 mainly lies on the type of partner. For example, LSD1, as a suppressor, can inactivate tumor suppressor genes via the activity of H3K4me2 demethylase. However, LSD1 can also promote transcription if it interacts with nuclear hormone receptors such as the androgen or estrogen receptors. Its enzymatic specificity switches to H3K9me2 [155, 156]. LSD1 is not restricted to histone and it is also able to demethylate other non-histones, such as p53 [157, 158].

Aberrant overexpression of LSD1 has been strongly correlated with poor prognosis in various kinds of human tumor types, such as hepatocellular, colon, breast, prostate, and non-small cell lung cancers [159–163]. Extensive studies have also demonstrated that LSD1 plays a crucial role in the early stages of carcinoma formation through chromatin remodeling [164, 165]. LSD1 can suppress p53-mediated apoptosis via demethylation of lysine-370 in p53 [158]. Previous reports have revealed that the expression of LSD1 is abundantly expressed in ovarian carcinoma tissues [166–169]. Moreover, one study observed that the level of LSD1 is improved from benign and borderline to malignant tumor in a stepwise manner, both in subtypes of serous and mucinous, and higher expression of LSD1 is linked strongly with FIGO stage and lymphatic metastasis in both ovarian serous cystadenocarcinoma and mucinous cystadenocarcinoma. Patients with a low level of LSD1 live longer than women with a high level of LSD1 expression [167]. A recent work shows that overexpression of LSD1 promotes ovarian carcinoma cell proliferation, migration, and invasion by regulating EMT. Knockdown of LSD1 impairs the ability of migration and invasion in ovarian cancer. Mechanistic analyses suggested that overexpression of LSD1 induces EMT and downregulates the transcription of E-cadherin which plays vital roles in regulating adhesion of cell-cell and maintenance of tissue architecture, with a concomitant upregulation of the mesenchymal markers (including N-cadherin, Vimentin and MMP-2). Furthermore, upregulated LSD1 inhibits the transcription of E-cadherin by demethylating H3K4me2 at the E-cadherin promoter. In addition, this study also observed that overexpressed LSD1 causes an increase in expression of transcription factor Snail that induces EMT through decreasing E-cadherin expression, and the loss of LSD1 leads to the downregulation of Snail [169]. Previous study showed that LSD1 is recruited through Snail to the region of E-cadherin promoter to inhibit the gene transcription of the E-cadherin consequently contributing to EMT-associated tumor cells invasion [170]. Sox2, a pluripotent stem cell (PSC) protein, is frequently expressed in many poor prognosis tumors and co-expressed with Oct4 and Lin28 in ovarian cancer [171, 172]. Sox2 serves as a pivotal regulator to confer certain stem cell properties to ovarian cancer cells to allow them to grow, differentiate and survive. It has been reported that the expression of Sox2 strongly depends on LSD1 expression [172, 173]. Downregulation of LSD1 represses the expression of Sox2 and induces cell-cycle arrest through directly increasing the methylation states of H3K4 and H3K9 on region of Sox2 and cell cycle genes. Strikingly, downregulation of LSD1 influences cellular differentiation through increase trimethylated H3K27. However, the upregulated level of trimethylated H3K27 is caused by the inhibition of Sox2 after the loss of LSD1, rather than by LSD1 inactivation directly [173]. Thus, LSD1 is a critical factor in ovarian carcinoma cell growth and differentiation via a Sox2-mediated histone demethylation mechanism.

Many researchers have confirmed that overexpression of epidermal growth factor receptor (EGFR) signaling is closely correlated with poor outcome of ovarian cancer [174–176]. Recently, a report indicated that the level of LSD1 increases in parallel with increased the level of EGFR in ovarian cancer. More importantly, the high level of LSD1 is induced by EGF signaling. Furthermore, EGF increases LSD1 expression by activation of the phosphatidylinositol 3-kinase (PI3K)/AKT signaling pathway, with a decrease of H3K4me2 [169]. LSD1 is a critical player in EGF-mediated ovarian cancer mechanism. These findings suggest that LSD1 holds considerable promise as a novel biomarker for diagnosis and a target for treatment in ovarian cancer.

#### KDM3 subfamily

The KDM3 family histone demethylases, including KDM3A, KDM3B and JMJD1C, are H3K9me2/me1 demethylases with a preference for dimethylated residues. They could remove the methyl groups from H3K9me2 to induce target gene expression activation [177]. They have been found to actively participate in the development and progression of a variety of cancers, including colorectal cancer [178], liver cancer [179], breast cancer [180] and Ewin sarcoma [181]. Recently, this superfamily has been found to regulate the malignant behaviors of ovarian cancers. Ramadoss et al. reported that KDM3A is a critical regulator of ovarian cancer stemness and cisplatin resistance [182]. KDM3A is crucial for the ovarian cancer cells to successfully progress through the critical stages of tumor progression such as cell proliferation, maintenance of CSCs and development of chemoresistance. To regulate these processes, KDM3A employs two distinct mechanisms; one by demethylating histone (H3K9me2) and the other by targeting a non-histone protein, p53. Mechanistically, while activating Sox2 expression by erasing the repressive methylation (H3K9me2) mark, KDM3A modulates p21 and Bcl-2 expression possibly through p53-K372me1 demethylation. Consistently, KDM3A depletion inhibited the growth of subcutaneously implanted cisplatin-resistant human ovarian cancer cells in athymic nude mice. Moreover, KDM3A is abundantly expressed and positively correlated with Sox2 expression in human ovarian cancer tissues. Thus, this report has unraveled a novel mechanism by which KDM3A promotes ovarian CSCs, proliferation and chemoresistance, underscoring the significance of KDM3A as a novel therapeutic target for resistant ovarian cancer.

#### KDM4 subfamily

Members of the KDM4/JMJD2 subfamily contain JMJD2A to JMJD2F. The KDM4/JMJD2 family is classified into two groups, based upon N-terminal JMJN and JMJC domain structure. JMJD2A, B, C belong to one group since their N-terminal JMJN and JMJC domain are followed by two PHD and two TUDOR domains [183]. The remaining members of the KDM4 subfamily (JMJD2E and JMJD2F) are sorted into a second group. JMJD2E and JMJD2F are currently regarded as pseudogenes because of a lack of intrinsic sequences in their structure [184]. Various studies have shown that KDM4 is upregulated in many tumors and is unequivocally needed for cancer cell proliferation [185]. Abnormal tumor cell growth, proliferation and blood vessels formation consume large amounts of energy and oxygen and further lead to severe hypoxic microenvironment. The hypoxic microenvironment in turn can contribute significantly to a number of human tumors [186], including ovarian carcinoma [187, 188]. The response to hypoxia in human tumors primarily are mediated by hypoxia-inducible factor (HIF) [186]. HIF is a heterodimer (including a α and a β subunits) and participates in a variety of tumor cell biological processes via inducing invasion, metastasis, angiogenesis, stem cell maintenance and resistance to chemotherapy and radiation. Accordingly, the expression of HIF is linked tightly with poor prognosis in human tumors [186, 189]. Multiple Jumonji-domain histone demethylases (JMJC-KDMs) can be regulated by HIF [190]. HIF-1α induces expression of several Jumonji-domain histone demethylases (JMJC-KDMs), including the KDM4 family [185]. The well-studied member of the KDM4 family is KDM4A/JMJD2A/JHDM3A. In general, the KDM4 family is suppressed in hypoxic microenvironment [185]. KDM4B/JMJD2B is thought to influence gene expression by demethylating di- and tri-methylated histone 3 at lysine 9 (H3K9me2/me3) and lysine 36 (H3K36me2/me3). Recently, Wilson et al. demonstrated that hypoxia-inducible histone demethylase KDM4B is upregulated in ovarian cancer, and the mechanism of KDM4B that regulates the expression of metastatic genes and pathways, and facilitates peritoneal seeding and growth of ovarian cancer cells through hypoxic signaling [185]. Moreover, the hypoxia-inducible KDM3A, which could demethylate H3K9me2/1 in hypoxic states, may promote the function of KDM4B in ovarian cancer [185]. Studies have shown that KDM4A is either a promotor or suppressor in gene post-transcription. KDM4A can compose complexes with either androgen or estrogen receptors and then activate these complexes’ activity through the KDM4A catalytic domain [191, 192]. It remains unclear whether the inhibition requires KDM4A enzymatic activity consistent with an oncogenic function of KDM4A. The overexpression of KDM4A in ovarian cancer has previously been established. It is stabilized by hypoxia, independent of HIF, to promote gene amplification, copy number heterogeneity gain and drug resistance in ovarian cancer [193, 194]. Further researches investigating the mechanisms mediated through the KDM4 family in ovarian cancer is still warranted.

#### KDM5 subfamily

KDM5 subfamily (also known as JARID1) is composed of four multidomain members: JARID1A (KDM5A/RBP2), JARID1B (KDM5B/PLU1), JARID1C (KDM5C/SMCX), and JARID1D (KDM5D/SMCY). This family (with the exception of JMJC and JMJN domains), are recognized by the existence of ARID DNA binding [195], C5HC2 zinc finger motif, and several histone-interacting PHD domains [196]. All KDM5 members are capable of removing methyl groups from di- and tri-methylated histone 3 at lysine 4 (H3K4me2/me3). In actively transcribed genes, this occurs at the starting region of transcription. Evidence suggests that the KDM5 subfamily acts as a driver in carcinogenesis [197]. KDM5A and KDM5B induces the growth of cancer cells, reduces the expression of tumor suppressor genes, facilitates the acquired tolerance of cancer-fighting drugs, and maintains tumor-initiating cells [198]. Recent data has shown that KDM5B exhibits frequent gain of function for alterations in ovarian cancer and the high level of KDM5B is closely associated with poor outcome and acquired drug resistance in malignant ovarian tumor [199]. KDM5B may act as a key biomarker to predict prognosis and acquired chemoresistance for patients with ovarian carcinoma. More interestingly, high KDM5A/B expression characterizes a small subpopulation of slowly cycling, tumor-initiating cells that are intrinsically resistant to a wide variety of cancer therapeutics, including both cytotoxic (e.g. Cis-platinum) and targeted agents (tyrosine kinase inhibitors, Bortezomib, B-raf inhibitors) [200–202]. Inhibition of KDM5A/B may prove useful in combination with conventional therapies to combat drug tolerance of ovarian cancer patients.

Although the previously described demethylases have been reported in ovarian cancer and implicated in tumorigenesis, the exact mechanism is not completely understood. It remains unclear whether the function of the remaining enzymes of demethylases are responsible for the onset and progression of ovarian carcinoma.

## Clinical applications

### Histone deacetyltransferase inhibitors (HDACis)

HDACis, as chromatin-modifying drugs, block HDACs and subsequently induces an increase in the acetylated level of histones. This stimulates the reactivation of silenced tumor suppressor genes and reverses the aberrant phenotype of malignant tumors. HDACis also induce differentiation of CSCs from their quiescent state. Other mechanisms of HDACis have also been identified, such as the generation of oxidative stress [203]. The inhibitors of histone deacetyltransferase represent a completely new insight into the therapy of malignant ovarian tumor and resistance to anti-cancer drugs. Several classes of HDACis have been identified, including organic hydroxamic acids, short-chain fatty acids, benzamides, cyclic tetrapeptides, and sulfonamide anilides [202]. Only three HDAC inhibitors have been approved by the FDA: vorinostat, romidepsin, and panobinostat. All three drugs have been successfully tested on ovarian cancer, either alone or in combination with other anti-cancer drugs such as cisplatin [204].

Many other HDACis are undergoing rapid development and have also been tested under preclinical and clinical investigation with potential to become anti-cancer drugs for ovarian cancer. For example, the HDAC inhibitor Trichostatin A (TSA), a drug that displays great inhibition for class I and II HDACs, can induce gene expression of P73 and facilitate Bax-dependent apoptosis in ovarian cancer cells with the acquired resistance of cisplatin [205]. Currently, TSA is still in the stage of preclinical stage for the treatment of ovarian cancer. Belinostat (Bel, PXD101) is a low molecular weight class I and II HDAC inhibitor of the hydroxamate class which alters acetylation levels of histone and non-histone proteins [206]. It has been investigated as a potent anti-tumor agent in a variety of cancers, including ovarian cancer. A Gynecologic Oncology Group (GOG) study was conducted to evaluate the impact of belinostat, in combination with carboplatin in women with platinum-resistant ovarian cancer. This study had 29 women enrolled and 27 were evaluable. The median number of cycles given was two (range 1–10). One patient had a complete response and one had a partial response, for an ORR of 7.4% (95% CI, .9–24.3%). Twelve patients had stable disease while eight had increasing disease. Response could not be assessed in five (18.5%). Grade 3 and 4 events occurring in more than 10% of treated patients were uncommon and limited to neutropenia (22.2%), thrombocytopenia (14.8%), and vomiting (11.1%). The median progression-free survival (PFS) was 3.3 months and overall survival was 13.7 months. PFS of at least 6 months was noted in 29.6% of patients. Due to the lack of drug activity, the study was closed after the first-stage [207]. Nevertheless, Dizon et al. further initiated a phase 1b/2 study was performed, with an exploratory phase 2 expansion planned specifically for women with recurrent EOC to evaluate the clinical activity of belinostat, carboplatin, and paclitaxel (BelCaP) [208]. Thirty-five women were treated on the phase 2 expansion cohort. BelCap was given as follows: belinostat, 1000 mg/m^2^ daily for 5 days with carboplatin, AUC 5; and paclitaxel, 175 mg/m^2^ given on day 3 of a 21-day cycle. The primary end point was overall response rate (ORR), using a Simon 2 stage design. The results showed that 54 % had received more than two prior platinum-based combinations, 16 patients (46%) had primary platinum-resistant disease, whereas 19 patients (54%) recurred within 6 months of their most recent platinum treatment. The median number of cycles of BelCaP administered was 6 (range, 1–23). Three patients had a complete response, and 12 had a partial response, for an ORR of 43% (95% confidence interval, 26–61%). When stratified by primary platinum status, the ORR was 44% among resistant patients and 63% among sensitive patients. The most common drug-related adverse events related to BelCaP were nausea (83%), fatigue (74%), vomiting (63%), alopecia (57%), and diarrhea (37%). With a median follow-up of 4 months (range, 0–23.3 months), 6-month progression-free survival is 48% (95% confidence interval, 31–66%). Median overall survival was not reached during study follow-up. The results showed that belinostat, carboplatin, and paclitaxel combined was reasonably well tolerated and demonstrated clinical benefit in heavily-pretreated patients with epithelial ovarian cancer. The addition of belinostat to this platinum-based regimen represents a novel approach to epithelial ovarian cancer therapy and warrants further exploration. Other information on the use of HDACis in ovarian cancer has been listed in Table 1. Although HDACis as a new class of anti-cancer drugs are quite frequently researched in the field of ovarian cancer, the underlying mechanisms remain unclear. Additionally, a huge issue concerning the development of HDACis in the therapy of human tumor (including ovarian cancer) is that most of them have severe side effects due to cytotoxicity to normal cells. Therefore, better selective inhibitors of HDAC should be explored for ovarian cancer treatment.

**Table 1:** Inhibitors of epigenetic modifications for the treatment of ovarian cancer

Classification		Drug	Target	Phase	Reference
HDACi	Hydroxamic acid	Trichostatin A(TSA)	Class I and II HDAC families	Preclinical	[214]
		Panobinostat(LBH589)	Class I and II HDAC families	Phase I	[215, 216]
		Belinostat(PXD101)	Class I and II HDAC families	Phase II	[207, 208]
	Short-chain fatty acid	Valproic acid(VPA)	Class I and IIa HDAC families	Phase I and Phase III	NCT00529022; NCT00533299
	Cyclic peptide	Romidepsin(FK288)	Class I HDAC family	Phase II	NCT00091195(Terminated); NCT00085527(Withdrawn)
	Benzamide	Mocetinostat(MGCD0103)	Class I HDAC family	Preclinical	[217]
HKMTi	S-adenosylhomocysteine hydrolase inhibitor	3-Deazaneplanocin A(DZNEP)	Polycomb group proteins	Preclinical	[209]
HDMTi	Polyamine analog	Polyamine analog	LSD1	Preclinical	[218]

### The inhibitors of HMTs or HDMTs

The balance between HMTs and HDMTs is required to keep the level stable for histone methylation. The imbalance between histone methylation and demethylation has been frequently found in ovarian carcinoma and is caused by mutations and aberrant gene expression. A number of inhibitors have been studied to target HMTs and HDMTs and are promising for ovarian cancer therapy. Development of histone methylation modulators is still in its preliminary stages. Currently, several inhibitors of HKMTs have been developed. 3-Deazaneplanocin A (DZNEP), the first indirect inhibitor of EZH2, was reported to be a promising cancer-fighting agent for malignant ovarian tumor, with potential to reduce proliferation, induce apoptosis, and inhibit metastasis [209]. More interestingly, Bitler et al. demonstrated that EZH2 inhibitor, GSK126, acts in a synthetic lethal manner in ARID1A-mutated ovarian cancer cells and that ARID1A mutational status correlated with response to the EZH2 inhibitor [210]. PIK3IP1 was identified as a direct target of ARID1A and EZH2 that is upregulated by EZH2 inhibition and contributed to the observed synthetic lethality by inhibiting PI3K–AKT signaling. Moreover, EZH2 inhibition caused regression of ARID1A-mutated ovarian tumors in vivo. Thus pharmacological inhibition of EZH2 represents a novel treatment strategy for ovarian cancers involving ARID1A mutations. In addition, a number of inhibitors of LSD1 also have been applied in ovarian cancer. However, most of them are non-selective to inhibit the activity of LSD1 [211]. Currently, some selective and potent LSD1 inhibitors have emerged [212], such as S2101. S2101 could suppress ovarian cancer cells via inducing autophagy and apoptosis. Additionally, the inhibition of AKT/mTOR signaling pathway also contributes to the anti-cancer effect of S2101 in ovarian cancer cells [213]. The use of nonselective compounds is restricted due to the undesirable side effects. Thus the synthesis of more selective derivatives needs to be discovered.

### Novel directions for epigenetic histone modification in the clinical management of ovarian cancer

As epigenetic inhibitors are an emerging therapy in ovarian cancer, and with development of more selective HDACi and perhaps targeting histotypes most likely to respond, this approach may find a way into clinical care. Moreover, with development of resistance to traditional chemotherapeutic regimens and the emerging immunotherapy, a combinatory treatment with epigenetic inhibitors might open a new avenue to fight against this deadly disease. Modern computational biology approaches will also guide the precision medicine therapy for the selection of epigenetic inhibitors for the treatment of specific histotypes of ovarian cancer. Studies of histone modification proteins are more nascent, yet clinical trials in inhibitors of these proteins are underway. Much more is needed to be done to fully realize the potential that epigenetics holds for ovarian cancer clinical care.

## Conclusion and perspective

Malignant tumor formation is associated with gene alteration and epigenetic change. Although genetic alterations cannot be reversed, epigenetic changes are reversible and thus are easy to modulate. Epigenetics may serve as the basis of the development of diagnostic tools aiding in early detection of malignant ovarian tumor. Aberrant histone acetylation and methylation alter gene expression, with potential clinical consequences of malignancy. In recent years, the study of histone modification and the involved regulatory enzymes have accelerated the identification of potential diagnostic and prognostic biomarkers for ovarian cancer. Epigenetic therapy, especially the modulators of histone-modifying enzymes, has been under the spotlight in ovarian cancer research. However, the comparative low specificity of these epigenetic drugs might lead to undesirable side effects to human, hampering its widespread clinical application. Modern scientist are endeavoring to discover novel generations of epigenetic drugs based upon the biological effects of this histone-modifying enzymes with maximal therapeutic efficacy and minimal toxicities. Although there is still a long way to go, epigenetics-based biomarker profiling and therapeutic regimens may provide a powerful weapon to fight against ovarian cancer.

## Abbreviations

ADMA: Asymmetric di-methylated arginine
AR: Androgen receptor
BRCA1: Breast cancer 1
CAFs: Cancer-associated fibroblasts
CSCs: Cancer stem cells
CTL: Cytotoxic T-cell
DOT1: Disruptor of telomeric silencing-1
DOT1L: Disruptor of telomeric silencing-1-like
DZNEP: 3-Deazaneplanocin A
EED: Embryonic ectoderm development
EGFR: Epidermal growth factor receptor
EMT: Epithelial-to-mesenchymal transition
EOC: Epithelial ovarian cancer
ER: Estrogen receptor
EZH2: Enhancer of zeste homologue 2
GNAT: GCN5-related N-acetyltransferases
GOG: Gynecologic Oncology Group
GPCRs: G-protein coupled receptors
HATs: Histone acetyltransferases
HDACs: Histone deacetylases
HDMTs: Histone demethylases
HE4: Human epididymis protein 4
HGSC: High grade serous carcinoma
HIF: Hypoxia-inducible factor
HLA: Human leukocyte antigen
HMTs: Histone methyltransferases
JMJD6: Jumonji domain–containing 6 protein
LPA: Lysophosphatidic acid
LSD1: Lysine-specific demethylase 1A
MEF2: Myocyte enhancer factor 2
MHC: Major histocompatibility complex
MMA: Mono-methylated arginine
NM23: Nonmetastatic 23
NSTCs: Non-stem tumor cells
ORR: Overall response rate
PD-L1: Programmed death ligand-1
PFS: Progression-free survival
PI3K: Phosphatidylinositol 3-kinase
PRC2: Polycomb repressive complex 2
PRMTs: Arginine methyltransferases
RGS2: Regulator of G-protein signaling 2
ROCA: Risk of ovarian cancer algorithm
SAM: S-Adenosyl-l-Methionine
SDMA: Symmetric di-methylated arginine
SIR2: Silent information regulator 2
SUZ12: Zinc finger protein suppressor of Zeste 12
TGF-beta1: Transforming growth factor-beta1
TILs: Tumor-infiltrating lymphocytes
TRX: Trithorax
TSA: Trichostatin A
